# Quantification of Inter-Sample Differences in T-Cell Receptor Repertoires Using Sequence-Based Information

**DOI:** 10.3389/fimmu.2017.01500

**Published:** 2017-11-15

**Authors:** Ryo Yokota, Yuki Kaminaga, Tetsuya J. Kobayashi

**Affiliations:** ^1^Institute of Industrial Science, The University of Tokyo, Tokyo, Japan; ^2^Department of Electrical Engineering and Information Systems, Graduate School of Engineering, The University of Tokyo, Tokyo, Japan; ^3^PRESTO, Japan Science and Technology Agency (JST), Saitama, Japan

**Keywords:** T cell, TCR repertoire, inter-repertoire comparison, pairwise sequence alignment, sequence dissimilarity, manifold learning, Jensen–Shannon divergence

## Abstract

Inter-sample comparisons of T-cell receptor (TCR) repertoires are crucial for gaining a better understanding of the immunological states determined by different collections of T cells from different donor sites, cell types, and genetic and pathological backgrounds. For quantitative comparison, most previous studies utilized conventional methods in ecology, which focus on TCR sequences that overlap between pairwise samples. Some recent studies attempted another approach that is categorized into Poisson abundance models using the abundance distribution of observed TCR sequences. However, these methods ignore the details of the measured sequences and are consequently unable to identify sub-repertoires that might have important contributions to the observed inter-sample differences. Moreover, the sparsity of sequence data due to the huge diversity of repertoires hampers the performance of these methods, especially when few overlapping sequences exist. In this paper, we propose a new approach for REpertoire COmparison in Low Dimensions (RECOLD) based on TCR sequence information, which can estimate the low-dimensional structure by embedding the pairwise sequence dissimilarities in high-dimensional sequence space. The inter-sample differences between repertoires are then quantified by information-theoretic measures among the distributions of data estimated in the embedded space. Using datasets of mouse and human TCR repertoires, we demonstrate that RECOLD can accurately identify the inter-sample hierarchical structures, which have a good correspondence with our intuitive understanding about sample conditions. Moreover, for the dataset of transgenic mice that have strong restrictions on the diversity of their repertoires, our estimated inter-sample structure was consistent with the structure estimated by previous methods based on abundance or overlapping sequence information. For the dataset of human healthy donors and Sézary syndrome patients, our method also showed robust estimation performance even under the condition of high sparsity in TCR sequences, while previous studies failed to estimate the structure. In addition, we identified the sequences that contribute to the pairwise-sample differences between the repertoires with the different genetic backgrounds of mice. Such identification of the sequences contributing to variation in immune cell repertoires may provide substantial insight for the development of new immunotherapies and vaccines.

## 1 Introduction

The development of high-throughput sequencing with next-generation sequencers has provided new opportunities to quantify T-cell receptor (TCR) repertoires and to compare their differences among different cell types, organisms, and pathological samples. Such information is indispensable for quantitatively understanding the immunological state of organisms that is shaped by the collection of immune cells. Moreover, the detailed information of TCR repertoires, especially that of inter-sample differences, is anticipated to significantly promote the development of immunotherapies and vaccines (1, 2). To this end, several statistical and computational methods have been proposed to quantify sample differences. One popular method is to quantify the fraction of overlapping sequences between two samples or among many samples such as the Bray–Curtis index (3–5). However, the possible sequence space of the TCR repertoire is at least as large as 10^15^ (6), and therefore the measured sequences can only sparsely cover the entire space. This sparsity substantially reduces the chance to observe exactly the same sequences in two samples. Thus, by focusing on the overlaps, it is only possible to detect the public sequences that appear very frequently among the samples. Moreover, even if no overlapping among the sequences is detected, it is not possible to judge whether this occurs because the two repertoires cover quite different subspaces of the sequence space or because the repertoires cover the same subspace but show no overlapping by chance simply owing to the sparsity of the coverage.

Another approach focuses on the count (abundance) distribution of unique TCR sequences in the repertoires (7). The methods based on the abundance distribution typically estimate the parameters of models of abundance distributions from experimental data and define the inter-sample difference according to the deviation of the estimated parameters. Poisson abundance (PA) models are among the recently developed methods based on the hierarchical Bayesian inference algorithm. This approach can overcome the substantial sampling fluctuations derived from the huge diversity in TCR repertoires and provides a stable result related to the inter-sample distances on the basis of statistical interpretations. Variations of PA models have also been proposed (8–10), and methods to combine both approaches were also developed recently. For example, Rempala et al. (11) used a bivariate Poisson log-normal (BPLN) distribution to model joint abundance distributions for classifying eight different samples of the following sample conditions: donor sites, types of T cells, and the genetic backgrounds of different mouse lines. Guindani et al. (12) also used a Poisson–Dirichlet process to classify types of T cells (i.e., conventional and regulatory T cells).

Although these PA models can successfully quantify the inter-sample distances, they are also associated with a major drawback in that some of the sequence information in the samples is lost since these models focus only on the count distribution. This loss of information has hampered the ability to determine the characteristic sequences of each sample, which is a requisite for further investigations of repertoires by, for example, evaluations of the interaction with microbial peptides (13) and the simulation of TCR crystal structures (14).

Moreover, the sparsity also affects some abundance-based methods in which the joint abundance distributions are employed.

To address these problems, we here propose a new dimensionality-reduction-based method for REpertoire COmparison in Low Dimensions (RECOLD): we focus on the sequence information in all samples and estimate the low-dimensional representation (manifold) by projecting and embedding the high-dimensional inter-sequence relations, calculated from pairwise sequence alignments, onto a low-dimensional space. The methods for manifold learning have been successfully applied in previous studies of virus evolution (15) and relationships of 16S rRNA gene sequences in bacterial genomes (16) to extract the evolutionary pathways and interconnections of bacteria. Recent studies in immunology have also employed embedding methods to visualize high-dimensional cytometry (17) and TCR repertoire (18, 19) data, although the embedding in these cases was mainly used only for visualization purposes. However, the low-dimensional embedding of the original sequences in repertoires contains information on how the repertoires from different samples cover the possible sequence space. Therefore, by employing such information, it may be possible to detect a subset of sequences in the repertoires that has a major contribution to the inter-sample difference.

To quantitatively compare the embedded sequences, we estimated a probability density function of the sequence distribution in the low-dimensional space. This density estimation compensates for the sampling bias due to unseen sequences from the sparsity of the measured sequences. Finally, we quantified the inter-sample differences between the estimated density functions of the individual samples using the Jensen–Shannon divergence (JSD). This information-theoretic measure characterizes the difference between two distributions by the probability of observing either one by chance with random sampling from their average. Thus, this measure can effectively and quantitatively capture information of two repertoires even with few overlapping sequences. By extracting the sequences that show a major contribution to the information-theoretic measures, the sequences most responsible for the inter-sample differences can be determined, which cannot be effectively identified with previous approaches.

The rest of the paper is organized as follows. We first describe the experimental data adopted to test our method and the step-by-step data analysis procedure, including (i) quantification of sequence dissimilarity with the pairwise sequence alignment algorithm, (ii) evaluations of four different manifold learning methods for projecting the sequence distribution in low-dimensional space, (iii) adoption of the kernel density estimation algorithm (KDE) to quantify the sequence distribution, and (iv) quantification of the inter-sample differences and identification of the major contributing sequences according to the JSD values of the distributions. To validate and demonstrate the applicability of our method, we adopt two datasets: one from mouse and the other from human TCR repertoires. In the dataset of transgenic mice, which has comparatively small diversity in TCR sequences, we verified that similar inter-sampling clustering can be obtained by both our method and previous methods despite their use of different modalities (sequence and count, respectively) of a repertoire. We further evaluated the statistical significance of our results using a bootstrap algorithm to confirm the derived inter-sample difference. In the clinical dataset of human TCR repertoires of Sézary syndrome patients, we demonstrate that our method can efficiently identify an inter-sample clustering structure even with the high diversity in the TCR repertoires, which hinders the previous methods from working. Overall, our method shows advantages over previous approaches in capturing more complete and quantitative information on TCR repertoires. This method is expected to be of value for understanding variation of the immunological states to facilitate development of immunotherapies and vaccines.

## 2 Materials and Methods

### 2.1 Sequence Data

In this study, we used two public datasets. The first is a dataset on mouse repertoires, which was published in Ref. (11). This dataset includes information of eight different TCR populations, which are classified according to donor sites, types of CD4^+^ T cells, and the genetic backgrounds of mice. The CD4^+^ T cells were collected and isolated either from the thymus or peripheral lymph nodes, which are labeled as “Thy” and “Per,” respectively. In addition, these cells were categorized into either naive T cells (TN) or regulatory T cells (TR) in accordance with the presence or absence of Foxp3 expression. The two genetic backgrounds of mice, which were labeled as “wild type (Wt)” and “Ep” in Ref. (11), commonly had strong restriction on rearrangement of V(D)J genes (i.e., the two *α*-chain rearrangements between J*α*2.6 and J*α*2 with a fixed V*α*2.9 segment and fixed *β*-chain V*β*14D*β*2J*β*2.6); this mouse line with TCR restriction was represented as TCR^mini^ in Ref. (20). The main difference between these mice is that the Ep mice were produced from the TCR^mini^ mice backcrossed with the mice that express transgenic class 2 major histocompatibility complex molecules bound to a single “Ep” peptide (20). Thus, Ep mice are expected to show a more restricted TCR repertoire than the TCR^mini^ Wt mice. To evaluate the diversity of the TCR repertoires, the complementarity determining region 3 (CDR3) of TCR*α* chains were sequenced and amplified. Further details on this dataset are described in Section 4 of Ref. (11).

We also analyzed a human TCR dataset that was published in Ref. (21). This dataset includes human TCR repertoires of six healthy donors (HD) and ten patients with Sézary syndrome (P), the latter of which were classified into four groups according to disease severities. The severity of Sézary syndrome is assessed by the proportion of the inflamed area relative to the entire skin surface (20, 50, 80, and 100%). Here, we used a part of this dataset related to the CDR3 sequences of TCR*α* chains derived from the peripheral blood T cells adopted from the two healthy donors and the ten patients. For the following two reasons, we used the data of only two healthy donors. One is that the TCR repertoires of three out of the six patients were sequenced using a different next-generation sequencing platform from that used on the other samples, including the patients. The other reason is the limitation of our computational resources. The more information about the donors is shown in Table S1 in Supplementary Material. The extraction of TCR clonotypes from raw FASTQ files was executed with MiXCR software (version 2.0) (22) using the default parameter values of functions. Since the number of sequenced reads correlates with the number of observed unique sequences, the bias in the numbers of “in-frame” reads among individual samples seriously affects the difference in their TCR repertoires. Therefore, we equalized the numbers of “in-frame” reads of individual samples to their minimum value using subsampling with a bootstrap algorithm. The procedure of this subsampling and further details on this dataset are described in Section 2.2.5 below and in the Methods of Ref. (21), respectively.

### 2.2 Data Analysis Procedure

**Figure 1:**
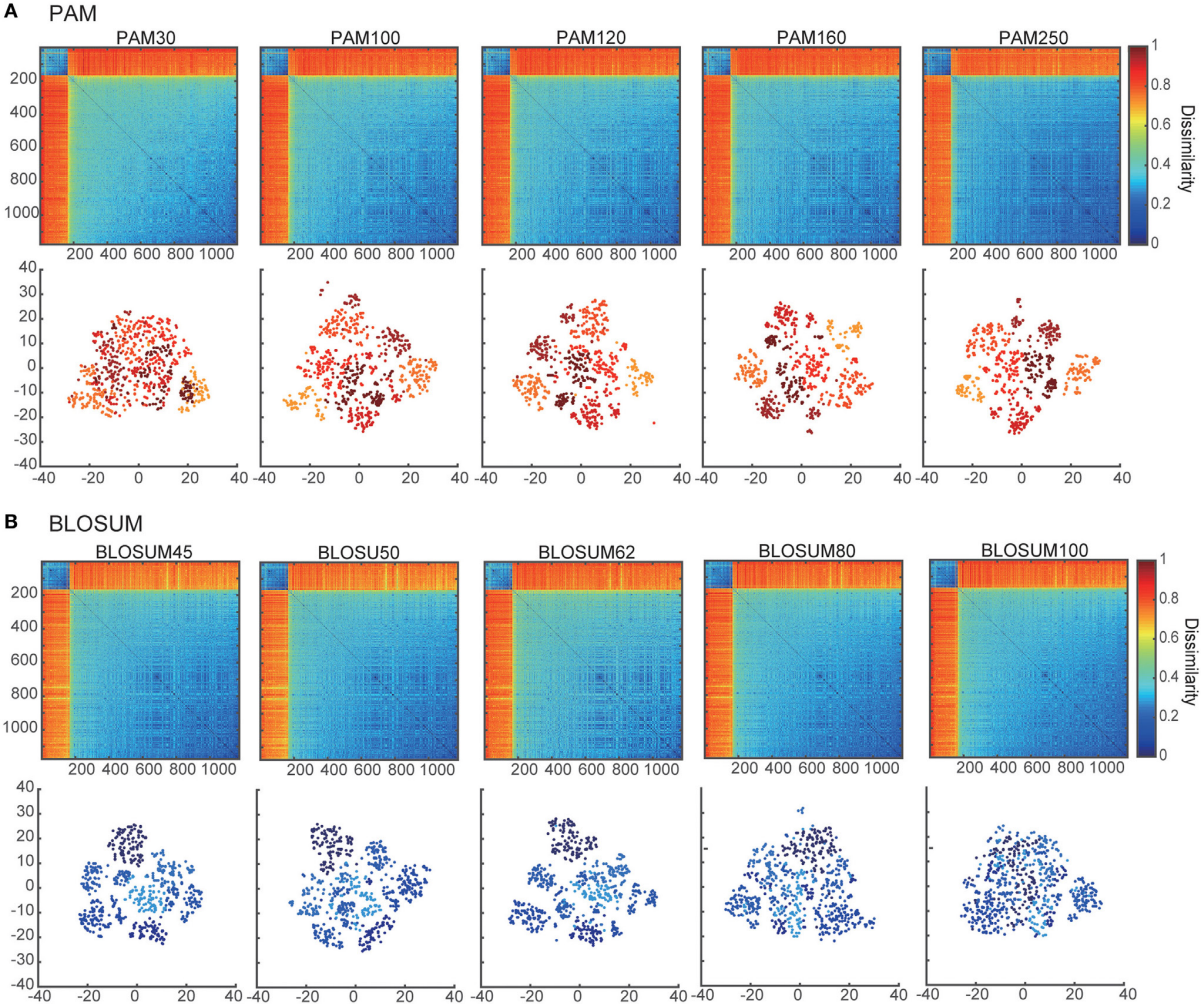
Dissimilarity matrices and their embedded distributions with 10 different score matrices: **(A)** PAM and **(B)** BLOSUM. The upper and lower panels show the dissimilarity matrices and projection maps in two-dimensional space, respectively. All of the rows and columns in each dissimilarity matrix were sorted according to the sum of their elements. The colors of points in the lower panels of **(A,B)** correspond to the clusters in PAM250 and BLOSUM45 that were discriminated by k-means algorithms (k = 7).

**Figure 2:**
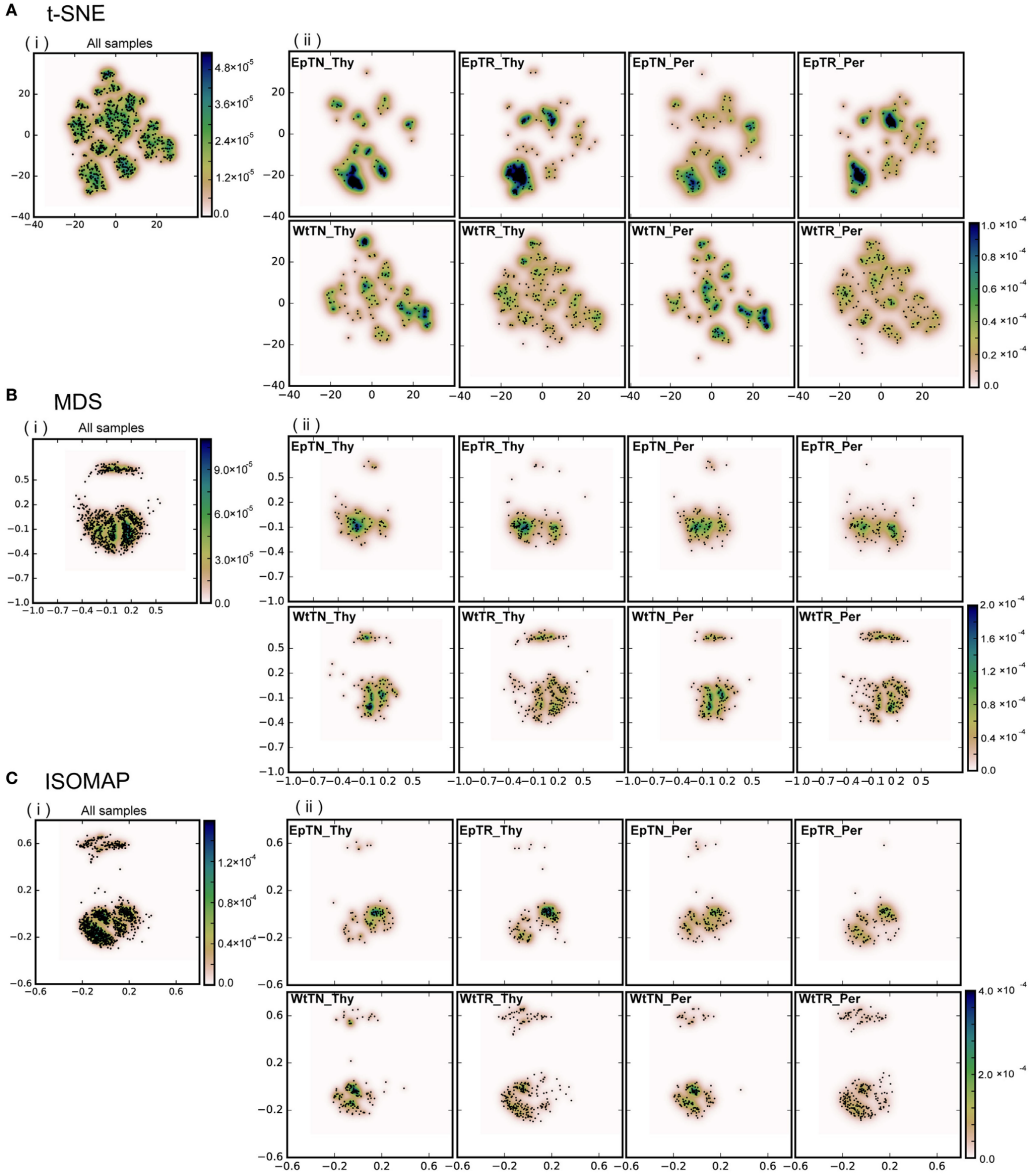

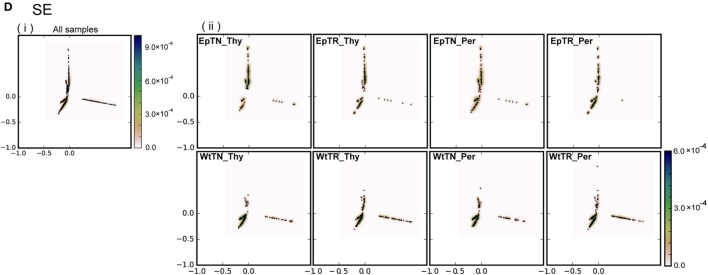
Dimensional reduction with four different dimensionality-reduction methods: **(A)** t-SNE, **(B)** MDS, **(C)** ISOMAP, and **(D)** SE. Panel (i) includes the points of the total unique sequences observed in all samples. Panel (ii) includes only the portions of sequences that were observed in each sample. “Ep” and “Wt” denote two different genetic backgrounds of mice. “TN” and “TR” denote naive and regulatory T cells. “Thy” and “Per” denote the thymus and peripheral lymph nodes, respectively. For example, EpTN-Thy denotes the naive T cells that were collected from the thymus in the “Ep” mice.

**Figure 3:**
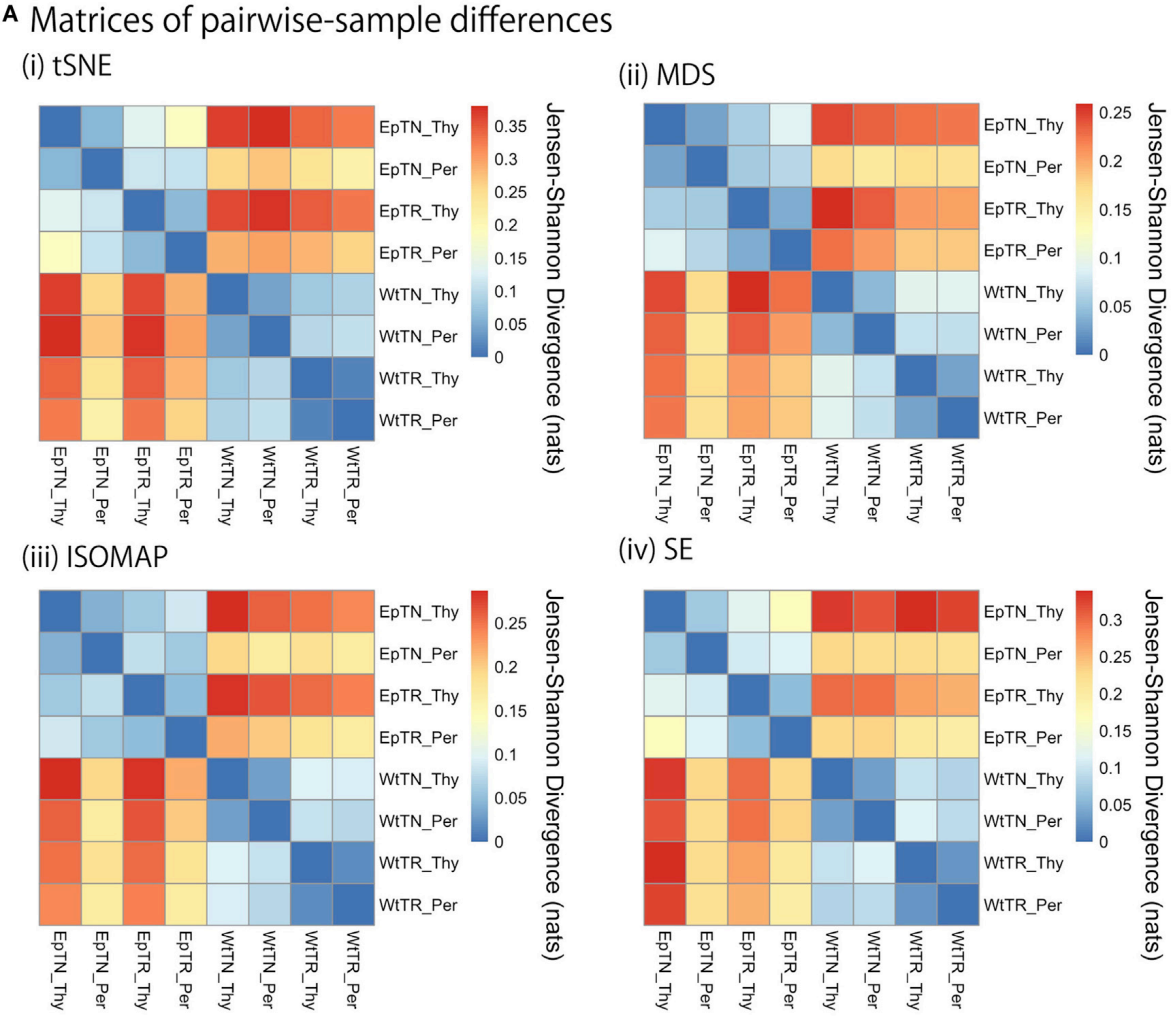

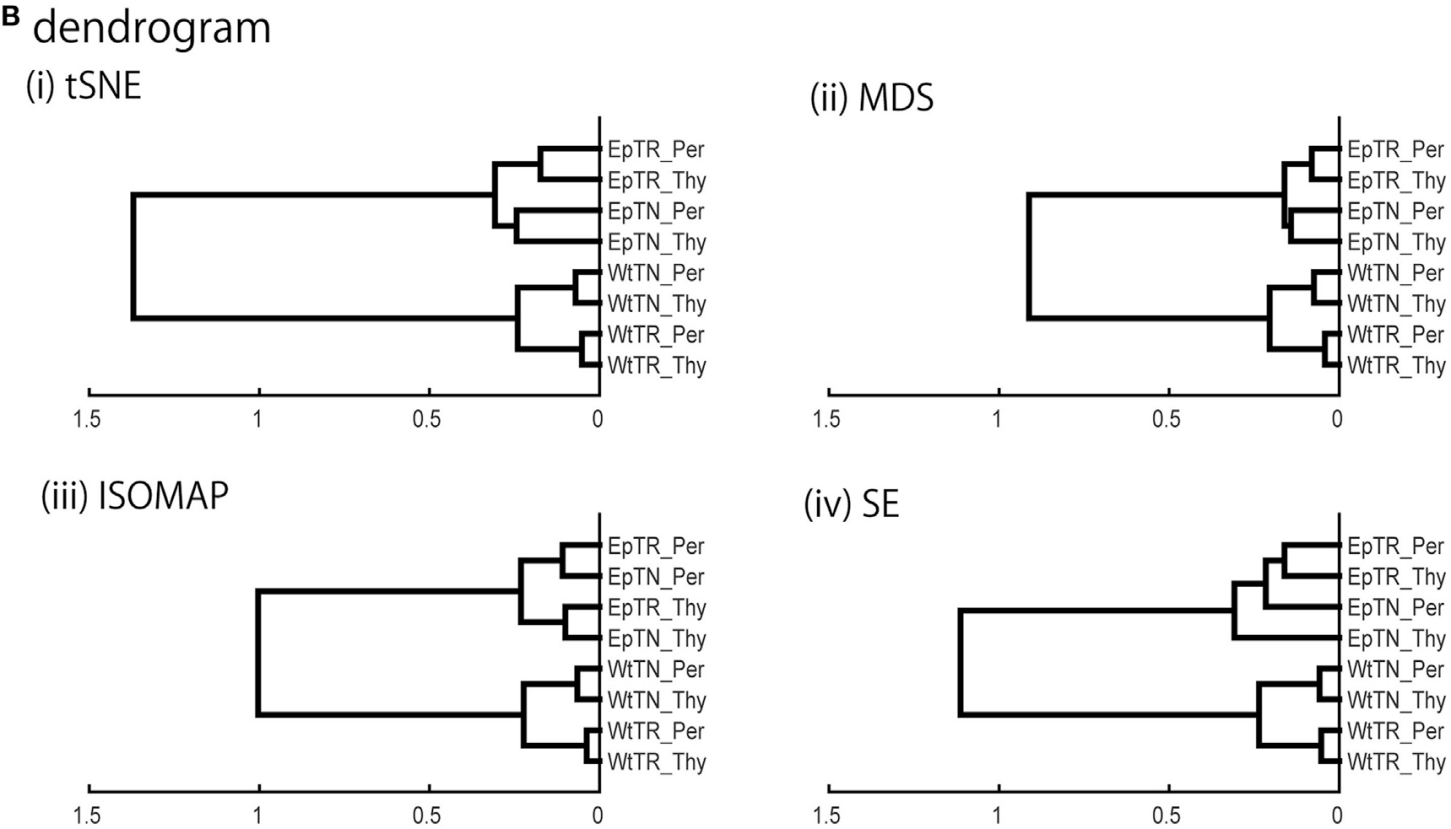
JSD matrices and their clustering results with four different methods: (i) t-SNE, (ii) MSD, (iii) ISOMAP, and (iv) SE. **(A)** Matrices of pairwise-sample differences and **(B)** the dendrogram constructed from the matrices.

To exploit detailed sequence information in repertoires and to circumvent the problem of diversity and sparsity, we focus on the sequence similarity among repertoires and derive its low-dimensional representation. Our method consists of five steps: (i) calculate a dissimilarity matrix of observed TCR sequences in all samples using the Smith–Waterman (SW) algorithm with a scoring matrix (Figure 1); (ii) embed the data in a low-dimensional Euclidian space by dimensionality-reduction methods while preserving the inter-sequence relations quantified by the dissimilarity matrix (Figure 2); (iii) estimate the sequence distributions in the low-dimensional space by the KDE algorithm (Figure 2); (iv) quantify the sample differences and cluster the samples by calculating the JSD value between the probabilistic density functions of different samples (Figure 3); and (v) identify subsequences that contribute to the differences (Figure 6; Table 1). Each of the above steps is described in detail in the following subsections.

**Table 1:** Sequences with high local JSD between EpTN-Thy and WtTN-Thy.

Regions	EpTN-Thy	WtTN-Thy
1	CAASAYQLIWG	CAARLYQLIWG
	CAASCYQLIWG	
	CAASRYQLIWG	
	CAASTYQLIWG	
	CAARNYQLIWG	
	CAAGNYQLIWG	
	CAAADYQLIWG	
	CAANNYQLIWG	
	CAASDYQLIWG	
	CAASNYQLIWG	
	CAATNYQLIWG	
	CAARDYQLIWG	
	CADSNYQLIWG	
	CAGSNYQLIWG	
	CAGGNYQLIWG	
	CASSNYQLIWG	
	CATSNYQLIWG	
	CAVSNYQLIWG	
	CGGSNYQLIWG	
	CVGSNYQLIWG	

2	CAARNYQLIWG	

3		CAAMDSNYQLIWG

4		CAAKDSNYQLIWG
		CAARDSNYQLIWG
		CAASDSNYQLIWG

5		CAASAWDSNYQLIWG

6		CAASNTGGLSGKLTFG

#### 2.2.1 Quantification of Sequence Dissimilarity

The first step of our method is the quantification of similarity for each pair of TCR sequences in all samples. The SW algorithm remains the most popular among pairwise local sequence alignment algorithms for quantifying the similarity of amino acid sequences (23). In recent years, improved versions of the SW algorithm have been proposed to resolve the problems related to the increase in computational costs along with the rapidly increasing size of datasets that are now possible from high-throughput sequencing. Here, we used the striped SW algorithm (24). This is one of the SW algorithm using a single-instruction-multiple-data (SIMD) system, which allows for multiple units to simultaneously execute the same operation. The algorithm was implemented with Parasail, an open-source software for sequence alignment (25).

The SW algorithm requires amino acid substitution matrices, which determine the cost of the replacement of a single amino acid residue by another (26). Although the SW algorithm has already been applied to TCR sequences as a mapping tool for CDR3 sequences (27), no study has yet established the best choice of substitution matrices for comparison of TCR data. Therefore, to clarify the effect of the type of substitution matrix employed and determine the optimal choice for our method, we tested 10 different matrices: five different point-accepted mutation matrices (PAM; 30, 100, 120, 160, and 250) (28) and five different blocks substitution matrices (BLOSUM; 45, 50, 62, 80, and 100) (26). The gap opening and extension penalties were set to 10 and 1, respectively (24).

Since the substitution matrices give non-zero values for replacements between the same amino acid residues, the total score of the alignment between two identical sequences depends on their sequence lengths. Thus, the diagonal elements of a pairwise distance matrix will have different values even when they are calculated from the alignments of two identical sequences. In other words, both the sequence similarity and the sequence length determine the values of the pairwise distance matrix. To adjust for this sequence-length effect, we converted the pairwise distance matrix into a dissimilarity matrix using the following equation:

(1) $$S_{i,j}=1-\frac{2D_{i,j}}{D_{i,i}+D_{j,j}}\;,$$

where *D_i,j_* and *S_i,j_* are a pairwise distance matrix and dissimilarity matrix between the two sequences *i* and *j*, respectively. At this step, we calculated the pairwise distances between all pairs of unique sequences observed in all samples with the striped SW algorithm. We then transformed the pairwise distance matrix into the dissimilarity matrix using equation (1).

#### 2.2.2 Dimensionality Reduction with Manifold Learning Methods

To visualize the structure of the high-dimensional dissimilarity matrix in a low-dimensional space, we applied dimensionality-reduction (manifold learning) techniques to the dissimilarity matrix described above that was constructed with BLOSUM62. Here, we compared the results calculated with four different methods: multidimensional scaling (MDS) (29), ISOMAP (30), spectral embedding (SE) (31), and t-distributed stochastic neighbor embedding (t-SNE) (32). All of these methods transform and embed the dissimilarity matrix *S* with dimensionality *N* into a new dataset *Y* with a lower dimensionality *d* in such a way as to preserve the structure of the dissimilarity matrix by minimizing cost functions. The major difference among these methods is the cost function, which is determined according to the relative distances between all pairs of sequences. MDS with a SMACOF algorithm minimizes the sum of squared errors in the relative distances of all sequence pairs before and after embedding (29). This cost function of MDS tends to preferentially retain the distances between more distant data points over those between more adjacent points (33). ISOMAP also minimizes the sum of squared errors, but rather than using the relative distances, it uses the geodesic distances, which are the distances along the shortest paths between two nodes on the neighborhood graph, calculated with a k-nearest neighbor algorithm (30). In the present study, we calculated the geodesic distances with the Warshall–Floyd algorithm (34). ISOMAP retains a neighborhood structure of data points lying on a curved manifold [e.g., the Swiss roll dataset (30)], which is collapsed in MDS. SE, also known as Laplacian eigenmaps, minimizes the cost function based on the neighborhood graph, which ensures that local neighborhood relations in a high-dimensional space are preserved in an embedded low-dimensional space (31, 35). We regarded the adjacency matrix based on the k-nearest neighbor algorithm as the weighted graph matrix to construct the Laplacian graph of SE. Finally, t-SNE converts the relative distances to joint probabilities, and minimizes the Kullback–Leibler divergence between the joint probabilities of the high-dimensional space and those of an embedded low-dimensional space (32). For calculation of the joint probabilities, t-SNE uses different kernels for the high- and low-dimensional spaces: a Gaussian kernel and a Student’s t-distribution, respectively. Since the Student’s t-distribution results in heavier tails than the Gaussian kernel, the t-SNE method emphasizes the local distances between data points in the low-dimensional space.

In studies of sequence alignments for sequences with different lengths, it is impossible to know the precise coordinates and the dimension of the sequence space. Thus, we cannot directly use principal components analysis, which is the most widely used dimensionality reduction technique (36) but requires vector data with fixed dimensionality. The common advantage of the above four methods is that if the distances between all pairs of data points are known, then there is no need to know the specific coordinates of the sequence space (32, 33).

We implemented t-SNE, MDS, and SE with the Scikit-learn manifold learning library (version 0.18.1) with Python (version 2.7.12) (37). ISOMAP was implemented with our custom-written code in Python, because the ISOMAP function of the Scikit-learn toolbox does not support the dissimilarity matrix as an argument. The detailed parameters of all methods are described in Table S2 in Supplementary Material.

#### 2.2.3 Estimation of the Probability Density Function with KDE

To compare the data points scattered in the embedded low-dimensional space among different samples, the embedded discrete data can be interpolated with a probability density function (PDF). Here, we estimated the PDF with the KDE algorithm (38–40). The exponential function was used as the kernel of the KDE (41). The bandwidth parameter of the exponential kernel function was optimized by maximum-likelihood estimation with a cross-validation algorithm (38). To reduce the computational cost of this calculation, we utilized the Kd-tree algorithm, which is an N-body algorithm that divides all of the data into N clusters based on their relative Euclidean distances (42). KDE was implemented with the parameter optimization toolbox in Scikit-learn (37). For application of the KDE, we discretized the embedded space with 400 bins along each axis with the following range: $[\text{min}x_{i}-(\text{max}x_{i}-\text{min}x_{i})/10,\text{max}x_{i}+(\text{max}x_{i}-\text{min}x_{i})/10],$ where *x_i_* indicates the position of a data point (i.e., a sequence) in the embedded space and *i* indicates each axis of that space.

#### 2.2.4 Quantification of Sample Differences with JSD

The final step of our method involves quantification of the inter-sample differences by calculating the JSD values between all pairs of the estimated PDFs (43). The JSD is defined as:

(2) DJS[P‖Q]=∫​DJSlocal(x)dx=∫12{P(x)logP(x)M(x)+Q(x)logQ(x)M(x)}dx=12DKL[P‖M]+12DKL[Q‖M] ,

where *P*(***x***) and *Q*(***x***) are the estimated PDFs and *D_KL_* is the Kullback–Leibler divergence; *M*(***x***) is $\frac{P(x)+Q(x)}{2}$ and $D_{JS}^{\text{local}}(x)$ is the “local JSD,” whose integration with respect to ***x*** gives the JSD. Thus, the “pairwise” JSDs provide an inter-sample distance matrix that quantifies the combinatorial differences between all pairs of the samples. To categorize all samples, we utilized hierarchical clustering, which converts the *N* × *N*-dimension sample-distance matrix into a dendrogram. Specifically, we used an agglomerative hierarchical clustering technique; each sample is initially treated as a singleton cluster, and pairs of clusters are repetitively merged according to a criterion until only a single cluster remains (44). We here used Wards criterion (45) for agglomerative clustering, which was implemented using the linkage function of Matlab’s Statistics and Machine Learning Toolbox (The MathWorks Inc., Natick, MA, USA). To compare our clustering result of the observed sequences with those obtained using other count-based methods, we also quantified the inter-sample difference with the BPLN and Bray–Curtis methods. BPLN was applied according to the methods described in the original paper by Ref. (11).

To evaluate the goodness of fit of the clustering results, Rempala and colleagues (11) calculated the cophenetic correlation coefficient (CCC), which quantifies the distortion due to the transformation from the distance matrix to the cophenetic matrix, from which the dendrogram was derived. However, the CCC does not always accurately reflect the goodness of fit of the results. Indeed, Wards method tends to produce lower CCC values than other methods such as average and centering methods, even though it was previously reported as the best agglomerative method (46, 47). Therefore, instead of the CCC, we verified the fit of the model based on the statistical significance of the distance between the nodes of the dendrogram, because the significance of the estimated value of JSD is unclear. Specifically, we used a bootstrap method to evaluate significance, resampled data points from the naive PDF according to the number of observed read counts, and then re-estimated the PDF from the resampled data points. We then calculated the JSDs between the naive and re-estimated PDFs. We repeated this process 100 times to obtain a histogram of the calculated JSDs. The 99th percentile of the histogram of the JSDs between the naive and each re-estimated PDF represents the one-sided confidence interval with 99% coverage, where values outside of the interval indicate a significance level of over 1%.

Finally, to identify the sequences with the greatest contributions to the inter-sample distances, we selected square bins for the top 1% of the local JSDs. We next defined the sequences in these bins as those contributing to the observed pairwise-sample difference. Furthermore, to investigate the characteristics of the contributing sequences, we calculated the relative frequencies of the amino acid residues in all of the contributing sequences of EpTN-Thy. The graphics of the relative frequencies were obtained using WebLog 3 software (48).

#### 2.2.5 Subsampling of CDR3 Sequences from Human TCR Repertoires

The following three steps were performed for subsampling CDR3 sequences of the human TCR repertoires in the dataset of Ref. (21). First, we calculated the cumulative relative frequency distribution *P*(*s*) of the observed unique sequences for each sample. Second, we generated random numbers from a uniform distribution between 0 and 1. The number of generated random numbers was equal to the minimum value of the “in-frame” reads among individual samples. Third, for each random number *x*, we selected a sequence *s* that corresponds to *s* = *P^−^*^1^(*x*). These selected sequences obtained from subsampling almost preserved the original relative frequency distribution, even though the number of “in-frame” reads are equalized among all samples. This subsampling procedure is indispensable for handling datasets that have a large bias in the number of observed sequences, because this bias will seriously affect the differences in TCR repertoires among samples.

All analyses were performed using custom-made codes written in Python (version 2.7.12), Matlab (R2015 a), and R (version 3.3.1).

## 3 Results

### 3.1 Evaluation of Sequence Dissimilarity for Pairwise Sequence Alignment

Using the pairwise sequence alignment and equation (1), which excludes the influence of the sequence lengths from the alignment results, we calculated the dissimilarity matrix of all pairwise sequences in the dataset of Ref. (11). The upper panels in Figures 1A,B show the dissimilarity matrices obtained with the 10 different substitution matrices, five of PAM and five of BLOSUM. As shown in these panels, the components of the dissimilarity matrices are clearly separated into two distinct clusters, which might reflect the *α*-chain rearrangements between J*α*2.6 and J*α*2 under the usage of the other fixed VJ genes. Moreover, the low-numbered PAMs and high-numbered BLOSUMs showed more gradual differences among the matrix elements than the others. This tendency was even more evident when viewing their embedded spaces for the separation of clusters. The lower panels in Figures 1A,B show the t-SNE projection maps of the corresponding dissimilarity matrices in the upper panels. In this case, low-numbered PAMs and high-numbered BLOSUMs tended to have merged clusters. This may be attributed to the specific characteristics of these two substitution matrices, which show higher variation in the scores for replacements between pairs of amino acids. Based on these results, we used the BLOSUM62 dissimilarity matrix for subsequent analyses for two main reasons. First, both the too high-numbered PAMs and too low-numbered BLOSUMs seemed to lose the intra-cluster structures by trying to compress the clusters into regions that were too small, while both the too low-numbered PAMs and too high-numbered BLOSUMs diminished any inter-cluster differences, resulting in indistinguishable clusters. Second, BLOSUM62 has been the most widely used matrix in analyses of TCRs and antigen peptides to date (13, 49–51).

### 3.2 Dimensionality Reduction of the Dissimilarity Matrix

To evaluate the applicability of dimensionality-reduction methods, we reduced the dimensionality of the dissimilarity matrix into a two-dimensional space using four different dimensionality-reduction methods (t-SNE, MDS, ISOMAP, and SE). In Figure 2, each point in each panel corresponds to a unique sequence of TCRs, and the spatial distances between pairs of points reflect the dissimilarity of the sequences corresponding to the points. In Figures 2A–D, panels (i) show the projection results of the unique sequences obtained from all samples, and panels (ii) show the subset of points (sequences) in (i) that appeared in the indicated sample (labeled in each panel), respectively. The differences of repertoires could be clearly reflected according to the scattering patterns of the points. Moreover, the points derived from the t-SNE and MDS methods spread more widely over the two-dimensional space than the others, whereas the points were more locally consolidated with the ISOMAP method, and especially with SE. This result suggests that t-SNE and MDS may be more appropriate than other reduction methods for larger datasets, because highly dense regions can cause difficulty in comparing the probabilistic distributions between samples. Furthermore, the two clear clusters in the dissimilarity matrix (the upper panel of BLOSUM62 in Figure 1B) were well reflected in the two clusters for the MDS and ISOMAP methods (Figures 2B,C), but were not represented clearly in the clusters of t-SNE. This result suggests that t-SNE emphasizes slight differences within clusters rather than large differences between the clusters of the dissimilarity matrix. Since it is unclear whether this visualization property of t-SNE works efficiently for comparisons between samples, we quantified and compared the distributions of data points at the next step and examined the method that would be most appropriate for this purpose.

### 3.3 Hierarchical Clustering of the Pairwise-Sample-Distance Matrix

We applied the KDE algorithms to the spatial distributions of data points to estimate their probability density functions (color gradient in Figure 2), for which JSD was calculated to quantify pairwise differences of repertoires. The matrices of the pairwise-sample differences are shown in Figure 3A, and the dendrograms in Figure 3B indicate the hierarchical clustering results with the agglomerative method. The clustering results can be categorized into two groups: ISOMAP and the others (MDS, t-SNE, and SE). The dendrograms of t-SNE, MDS, and SE showed a consistent hierarchical relation with the experimental conditions, in which the samples were ranked in order of donor sites, types of T cells, and genetic background with clear biological significance (11). By contrast, the dendrogram of ISOMAP showed a mismatch in the hierarchical order between the T-cell types and donor sites of Ep mice.

**Figure 4:**
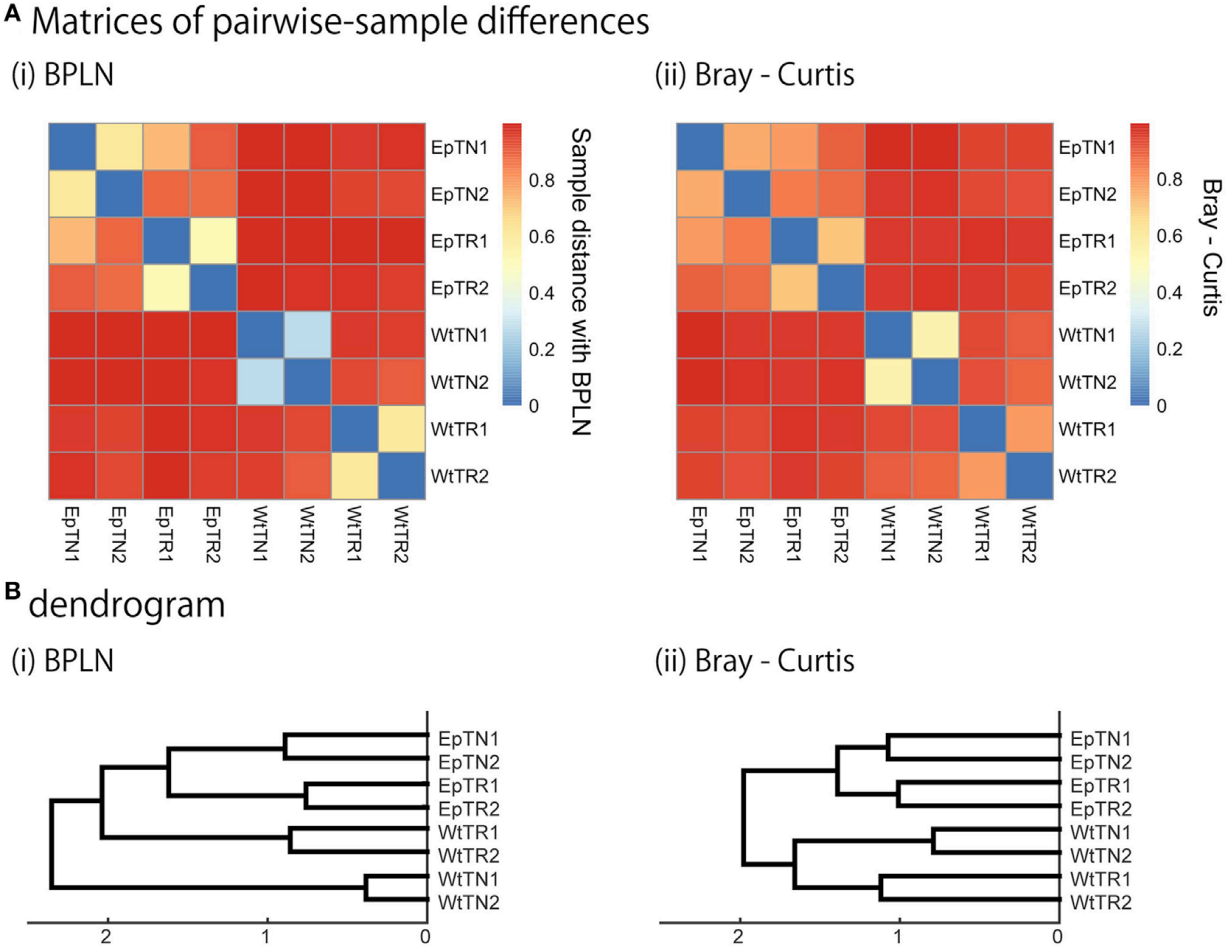
Sample-distance matrices constructed with two methods: (i) BPLN, (ii) Bray–Curtis. **(A)** Matrices of pairwise-sample distances and **(B)** the dendrogram constructed from the matrices.

To verify the relevance of the hierarchical clustering obtained by our method, they were compared with those obtained with previous count-based methods, the BPLN method, and the Bray–Curtis method. As shown in Figure 4, the sample differences and dendrograms estimated from the BPLN and Bray–Curtis methods were very similar to those obtained using our approach with MDS, t-SNE, and SE. Importantly, these similar results were obtained with different data modalities: sequence similarity in our method and observation counts in previous ones. Therefore, this consistency suggests that there is common information between sequence similarity and observation counts with respect to quantifying the differences among samples. We should note that these two modalities can be combined simply by assigning the number of observed sequence counts as a weighting factor for each data point (i.e., a unique sequence) in the embedded space. Indeed, the counts-weighted PDFs using KDE (Figure S1 in Supplementary Material) showed no obvious change in the hierarchical clustering structure of the pairwise-sample differences. Taking these results together, the MDS or t-SNE appears to be the better choice as a dimensionality-reduction method for evaluation of differences in TCR repertories among samples, given that these methods show wide spatial distributions of the data points and also show the most consistent dendrogram structures with those of previous count-based methods.

### 3.4 Significance Test for Inter-Sample Differences

**Figure 5:**
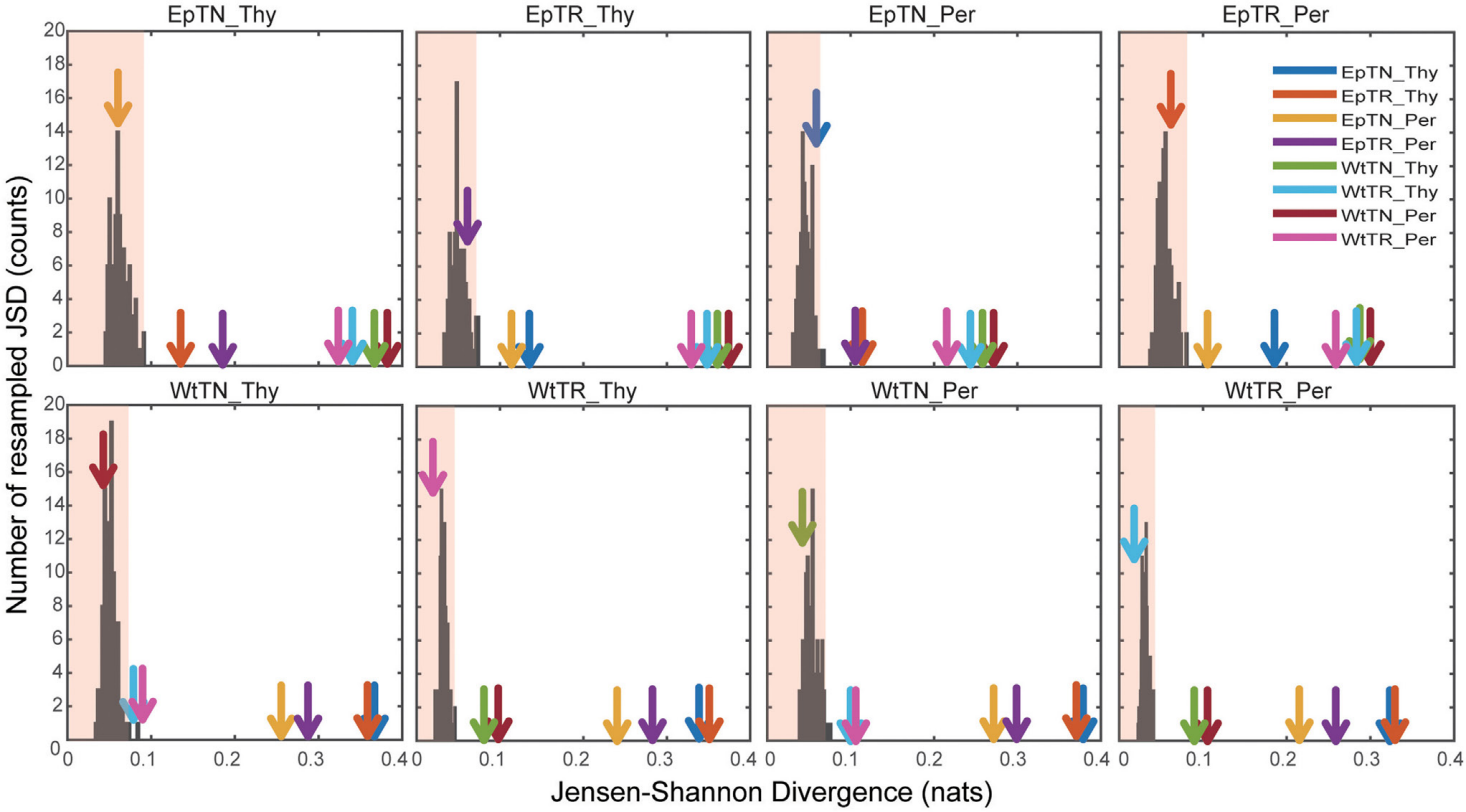
Significance tests of JSD values using bootstraps. Each colored arrow indicates the naive JSD values of Figure 3A(i). The light red region indicates the one-sided confidence interval with 99% coverage.

To verify the statistical significance of the calculated JSDs between all sample pairs, we calculated the JSDs between the naive and the re-estimated PDFs using a non-parametric bootstrap algorithm. Figure 5 shows the histograms of the JSD values between the naive PDF of Figure 2A, ii, and the re-estimated PDFs. In the figure, arrows indicate the naive JSD values between the sample designated on the top of the panel and the other samples. If the values indicated by the arrows are bigger than the light red region in the panel, the pairwise naive PDFs deriving the naive JSD are significantly different to each other. The arrows that indicate the JSD values between the pairs in proximity to the terminal nodes of the dendrogram in Figure 3A, i, were all within the light red regions, which means that these JSD values were not significantly bigger than those of the histogram. Since these pairs correspond to the difference of donor sites, this result suggests that the PDFs of these repertoires from different sites are so similar that they cannot be statistically distinguished from each other. By contrast, the arrows that indicate the naive JSD values between pairs in the upper parts of the clusters, above the terminal nodes, were outside of the light red regions, which means that the JSD values were significantly different from each other. This result indicates that the types of T cells and the genetic background can be discriminated with sufficient statistical significance.

### 3.5 Spatial Distribution of Local JSD Values

**Figure 6:**
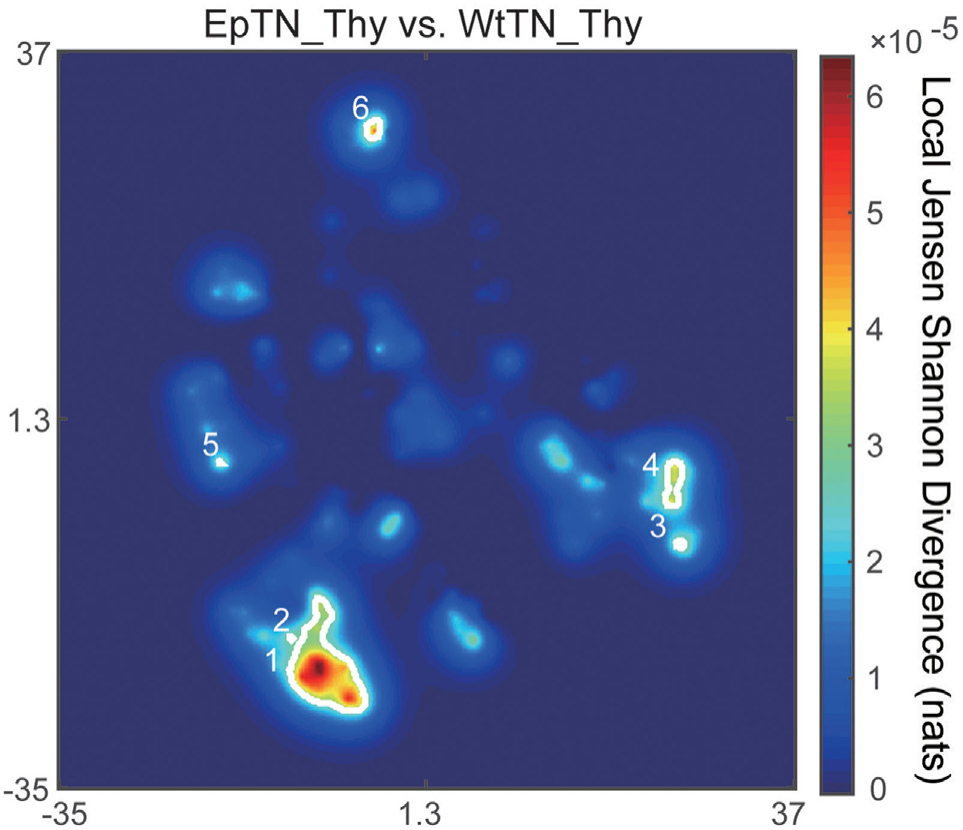
Spatial distribution of the local JSD values between EpTN-Thy and WtTN-Thy. The white curves show the contours of the regions with significantly high local JSDs.

The main advantage of our method compared to count-based methods is the ability to identify the major sequences contributing to inter-sample differences. To identify the sequences with the greatest contributions of local JSD to JSD values, we plotted the spatial distribution of the local JSDs between the WtTN-Thy and EpTN-Thy sequences. As shown in Figure 6, six regions were identified that were associated with the top 1% significance values. Table 1 lists the identified sequences in these regions with larger local JSDs than the others. In regions 1 and 2, there was only one sequence for WtTN-Thy, whereas EpTN-Thy had multiple sequences in these regions. By contrast, the regions 3, 4, 5, and 6 had several sequences of EpTN-Thy, whereas they had no sequence of WtTN-Thy. This result suggests that these unilaterally observed sequences may contribute to the observed abnormality in the antigen presentation of Ep mice.

**Figure 7:**
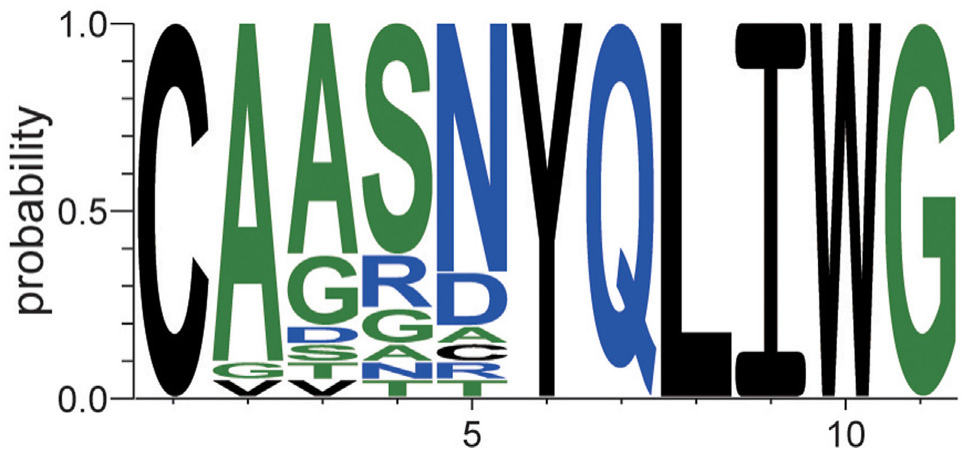
Relative frequencies of observed amino acids at each position in the contributing sequences of EpTN-Thy.

This type of sequence identification can provide further knowledge about the characteristics of the sequences. Figure 7 shows the occupation probability (relative frequency) of amino acids at each position of the sequences, which were observed only in EpTN-Thy and contribute to pairwise differences. This result suggests that a consensus sequence was determined from the 6th to 11th amino acid position. We note that these contributing sequences and their characteristics cannot be easily identified simply by examining the overlapping sequences in two samples, because there was almost no overlap between EpTN-Thy and WtTN-Thy sequences (0.352%, 1/284) and because these contributing sequences account for only 7.39% (21/284) of the total unique sequences in the two samples.

### 3.6 Application to Human TCR Repertoires

**Figure 8:**
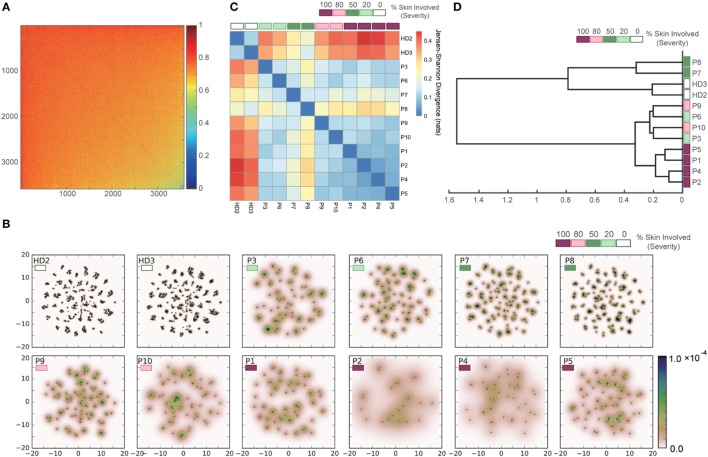
Results of applying our methods to the dataset of the human TCR *α*-chain CDR3 sequences derived from the peripheral blood T cells adopted from the two healthy donors (HD2 and HD3) and the ten Sézary syndrome patients (P1–P10). **(A)** Dissimilarity matrix of resampled sequences in all samples. **(B)** Embedding results of the dissimilarity matrix in **(A)** by t-SNE, and estimated PDFs by the KDE algorithm. **(C)** Sample-distance matrix with the PDFs in **(B)**. **(D)** The dendrogram constructed from the matrix in **(C)**.

To validate the applicability of our approach to datasets with greater diversity and sparsity, which are typical of clinical human TCR repertoires, we applied the same procedure to the dataset of Ref. (21) for human TCR repertoires of patients with Sézary syndrome. Figure 8A shows the pairwise dissimilarity matrix of unique sequences among all samples. The number of “in-frame” reads of each sample was equalized by subsampling. Since this dataset has no restriction in available V(D)J genes for recombination, it appears that there is no obvious cluster in the dissimilarity matrix. As shown in Figure 8B, the projection results of this dissimilarity matrix of Figure 8A indicate that the clusters observed in the point distributions of the healthy donors became less consolidated according to the severity of Sézary syndrome. The pairwise-sample differences in these point distributions were quantified and shown in Figure 8C, and the samples are hierarchically clustered in Figure 8D. These results indicate that our method almost successfully captures a hierarchical relation among the samples with respect to the severity of Sézary syndrome. The failure of the hierarchical clustering in the samples with 20% severity might be attributed to the abnormality of sample P3, which has an extremely low number of “in-frame” reads (Figure S3 in Supplementary Material). Moreover, the methods based on observation counts (e.g., BPLN and Bray–Curtis methods) cannot estimate the plausible hierarchical structures (Figure S2B in Supplementary Material) because of the rareness of overlapping sequences due to the high diversity and sparsity of the normal repertoires. Indeed, Bolkhovskaya et al. (52) also stated concerns related to BPLN because of this vulnerability in the face of the high diversity of normal TCR repertoires. Overall, these results suggest that our method can estimate the inter-sample hierarchical structure more robustly than the previous methods by overcoming the high diversity and sparsity problem in human TCR repertoires.

## 4 Discussion

We quantified the difference in TCR repertoires among different samples based on amino acid sequence dissimilarity. Through a quantitative comparison of the sequence distributions in the low-dimensional-embedded spaces of the dissimilarity matrix, we estimated an inter-sample hierarchical structure. For the restricted TCR repertoires of the transgenic mice, we demonstrated that our estimated structure was almost identical to that estimated with previous count-based methods that did not incorporate detailed sequence information. Furthermore, we identified the sequences that contribute most strongly to the pairwise-sample difference using the local JSD distribution. To validate our method for human TCR repertoires with much higher diversity and sparsity in TCR sequences than those of the transgenic mice, we confirmed that the estimated hierarchical clustering structure has a good correspondence with the severity of Sézary syndrome.

Despite the fact that our method relies on sequence similarity and previous methods are based on observation counts, which are completely different features of the TCR repertoire, almost the same clustering structure among samples was obtained in the dataset of the transgenic mice. This suggests that there is a relationship between the observation counts and sequence similarities, which was further confirmed by the lack of obvious change in the structure of the hierarchical clustering when estimated by taking observed sequence counts into account as weights (Figure S1 in Supplementary Material). Further studies to understand this relation in greater depth and generality would allow for cross-checking the results of sample classification by investigating the consistency of the two methods. Moreover, more detailed classification of repertoires may be possible by clearly distinguishing the overlapping and non-overlapping information between counts and sequences.

Although our method and the counts-based methods provide similar classification results, there are three merits of our method. The first is the robustness against errors derived from polymerase chain reaction (PCR) amplification bias attributed to the variability in reproducibility for individual sequences (53). Previous studies have shown that the PCR efficiency is affected by sequence profiles such as the length and GC content (54, 55). Indeed, high-throughput sequencing with DNA barcoding has confirmed that the PCR amplification efficiency of TCR sequences is highly variable due to the differences in profiles of individual cDNA molecules (27, 56, 57). This fact suggests that the PCR process for TCR sequence amplification induces errors in the numbers of observed sequence counts, which may eventually lead to errors in the results of counts-based methods such as PA models. Alternatively, our method does not depend on the sequence counts, allowing for reliability against errors due to PCR bias. The second key merit of our method is the ability to identify the sequences with the greatest contributions to pairwise-sample differences. This sequence identification allows for targeted analyses along with the results of other studies such as the simulation modeling for determining the crystal structures of the TCRs encoded by these sequences (14) or establishing alignments between CDR3 sequences and microbial genomes (13). Such a closed-loop experimental design may help to achieve a breakthrough in the development of vaccines or immunotherapies (1). The last merit is robustness to high diversity and sparsity. As the diversity and sparsity of the repertoires increase, the overlapping sequences, which the multivariate PA models and conventional methods in ecology rely on, are less likely to be observed. This trend makes the behaviors of these methods unstable for highly diverse repertoires. By contrast, since our method does not focus on the overlapping of sequences but rather on the low-dimensional representation derived from the dissimilarity of observed sequences, the decrease in the number of overlapping sequences in highly diverse repertoires may have much less of an impact on the performance of our method. In summary, these results and advantages demonstrate the potential applicability of adopting a sequence-based method in repertoire analysis, which can compensate for the drawbacks of conventional count-based methods.

Nevertheless, there are several issues and problems that should be mentioned that are worthy of further investigation for the development and improvement of sequence-based approaches for the comparison of TCR repertoires.

One issue concerns the treatment of gap penalties. When we evaluated the differences of the score matrices shown in Figure 1, we fixed the gap opening and extension penalties to 10 and 1, respectively. Although the effects of the penalties have not been adequately investigated in previous studies (58), the gap opening penalty was found to affect estimations of the hierarchical clustering structure (data not shown). The CDR3 region of TCR is a much shorter sequence than peptide sequences and also shows frequent deletions and insertions from somatic recombination events. Considering these characteristics, further investigations about the effects of gap penalties are needed. In addition, in terms of sequence alignment, we should consider the introduction of recently proposed novel substitution matrices taking into account the affinity against MHC (59) and specific epitopes (18). Since these matrices are specialized for TCR properties, they may provide more clues into the differences in TCR repertoires among samples.

The second aspect worthy of further consideration is the dependency of the results on the random numbers used for optimization and the toolboxes used for implementation. As shown in Figures S4–S9 in Supplementary Material, the previous version (version 0.16.1) of the Scikit-learn toolbox showed subtly different results, although this difference did not affect our conclusions or arguments. The main cause of this difference may be the change of the stopping criteria of iterations for manifold learning methods. Moreover, even when using the same toolbox (with the same version), the calculated results can be slightly different depending on the random numbers used in optimization.

The third issue is the empirical nature of the cost functions used in the dimensionality-reduction methods. As demonstrated in Figure 2, the scattering patterns of the sequence data in the low-dimensional space depend on the cost functions of the method adopted. Using MDS and ISOMAP, we obtained two clear clusters reflecting two regions in the dissimilarity matrix. This is because the cost function of MDS preferentially preserves the distances between the distant points rather than those between nearby points (33). By contrast, t-SNE emphasizes the local structures of nearby points over global points by using the Student’s t-distribution as the kernel of the embedded space. These cost functions were empirically determined for visualization purposes in the original papers, without consideration of the subsequent quantitative inter-sample comparison of the embedded results. Although our results suggest that the empirical combination of dimensionality-reduction methods and comparison of the embedded results by JSDs may work well, both the projection method and comparison methods in the embedded space should be consistently designed so as to best reflect the inter-sample difference in the original sequence space. This method might be developed by choosing an information-theoretic measure for the cost function of projection that can preserve the relevant information of repertoires in the original sequence space. Because the underlying high-dimensional structures of the repertoire are difficult to capture intuitively, methods based on firm theoretical rationality and biological significance are indispensable for further exploitation of repertoire information.

## Author Contributions

RY, YK, and TK: study conception and design; RY and YK: performed the research; RY and YK: data analysis; and RY and TK: wrote the paper.

## Conflict of Interest Statement

The authors declare that the research was conducted in the absence of any commercial or financial relationships that could be construed as a potential conflict of interest.
