# Supplementary material for: Quantification of Inter-Sample Differences in T-Cell Receptor Repertoires Using Sequence-Based Information
Source: Front Immunol. 2017 Nov 15;8:1500. doi: 10.3389/fimmu.2017.01500 (PMC5694755; doi:10.3389/fimmu.2017.01500)
Supplement: Supplementary file 1 [file Presentation_1.PDF]

Supplementary Material: Quantification of inter-sample differences in T cell receptor repertoires using a sequence-based information

Ryo Yokota*, Yuki Kaminaga and Tetsuya J. Kobayashi
*Correspondence:
Ryo Yokota
yokota@sat.t.u-tokyo.ac.jp

1 ESTIMATION OF COUNT-WEIGHTED PROBABILITY DENSITY FUNCTIONS

The method of estimating the sequence distribution weighted by the observation count of each sequence follows the same procedure. The only difference from the procedure in the main text is to insert the following step before estimating PDF by KDE; that is to pile the points at exactly the same position of each unique sequence as many times as we observed. Therefore, the estimated PDF has a large value in the position of each sequence in proportion to its observation counts. The results of this count-weighted method are shown in Fig.S1.

2 DEPENDENCY OF THE RESULTS ON THE VERSION OF SCIKIT-LEARN TOOLBOXES

In Figs.S4-9, we show the results obtained with the same data as Figs. 1 and 2(A), but using the version 0.16.1 of the Scikit-learn toolbox instead of 0.18.1 as in the main text. The major change between these versions may be modification of the stopping criteria for iterations in the t-SNE code. Depending on the choice of version, the results of our study can be slightly changed, but the change is small enough not to affect our conclusions or arguments.

3 SUPPLEMENTARY TABLES AND FIGURES

Supplementary Table 1. The detail of all donors (2 healthy donors and 10 patients) and labels used in the main text.

Severity	0%(Healthy)	20%	50%	80%	100%
ID of donors	HD2, HD3	P3, P6	P7, P8	P9, P10	P1, P2, P4, P5

Supplementary Table 2. Detailed parameters of manifold learning methods.
Except for ISOMAP, we used the functions in the class of sklearn.manifold in the scikit-learn toolbox. For ISOMAP, we first calculated the geodesic distances using the k-nearest neighbor algorithm and Floyd-Warshall method. We then applied the geodesic distances to MDS with the indicated parameters.

tSNE	parameters	value
	n_components	2
	random_state	0
	metric	'precomputed'
	the other parameters	default
MDS	parameters	value
	n_components	2
	maximum iteration	400
	relative tolerance w.r.t stress to declare converge	1.00E-5
	dissimilarity	'precomputed'
	the other parameters	default
SE	parameters	value
	n_components	2
	n_neighbors	50
	affinity	'precomputed'
	the other parameters	default
ISOMAP	parameters	value
	n_components	2
	maximum iteration	1000
	relative tolerance w.r.t stress to declare converge	1.00E-5
	metric	'precomputed'
	n_neighbors	50
	the other parameters	default

Supplementary Table 3. Sequences with high local JSD between EpTN-Thy and WtTN-Thy.

Regions	EpTN-Thy	WtTN-Thy
1	CAASAYQLIWG<br>CAASCYQLIWG<br>CAASRYQLIWG<br>CAASTYQLIWG	
2	CAAGNYQLIWG<br>CAAHNYQLIWG<br>CAANNYQLIWG<br>CAARNYQLIWG<br>CAASNYQLIWG<br>CAATNYQLIWG<br>CADLNYQLIWG<br>CADSNYQLIWG<br>CAGSNYQLIWG<br>CASHNYQLIWG<br>CASSNYQLIWG<br>CATSNYQLIWG<br>CAVSNYQLIWG<br>CVGSNYQLIWG	

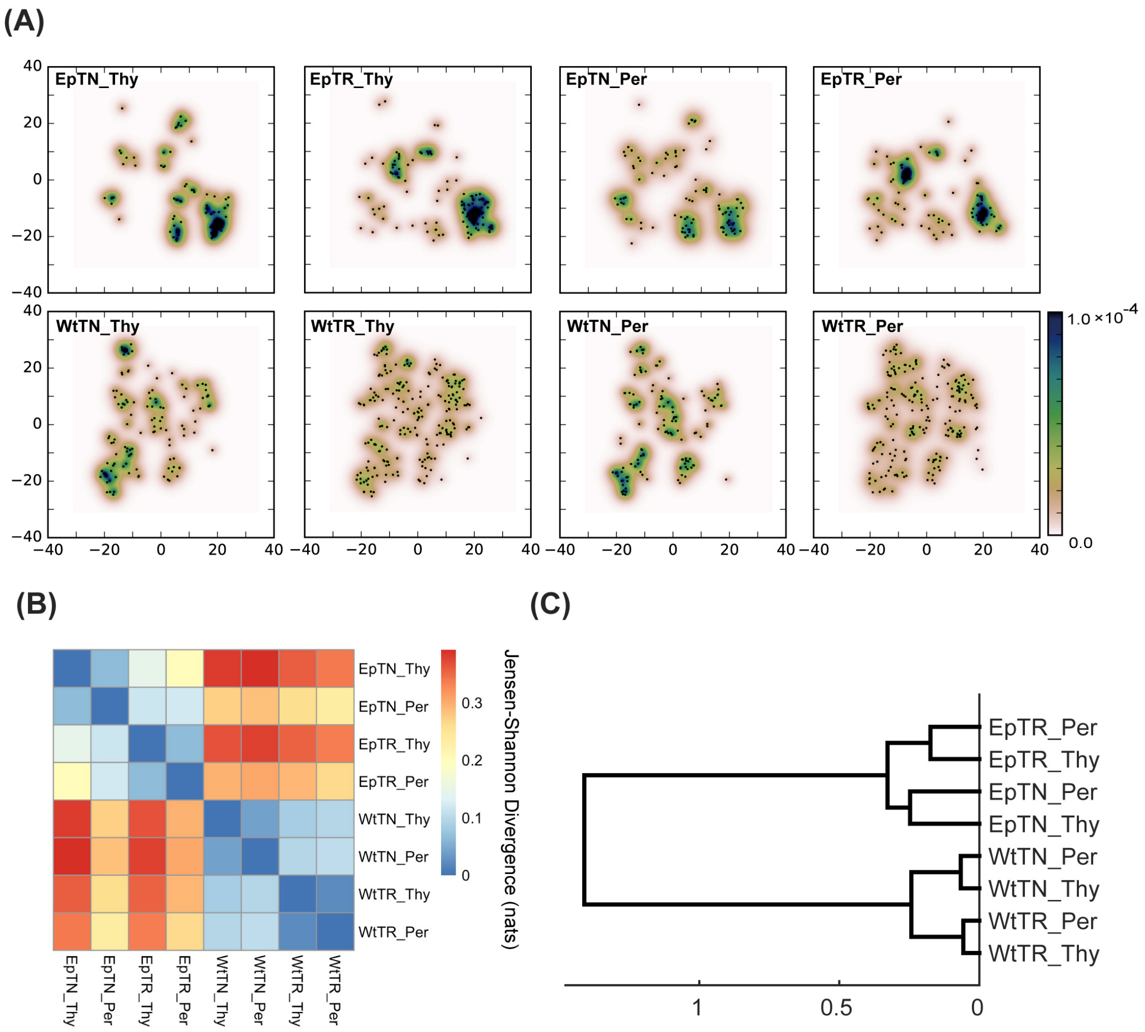

Supplementary Figure 1. Count-weighted PDFs estimated with tSNE-embedding data.
Labels correspond to those in Figs. 2 and 3.
(A) PDFs weighted by the number of sequence counts. (B) Sample distance matrix estimated with the weighted PDFs. (C) Dendrogram constructed from the matrix in (B).

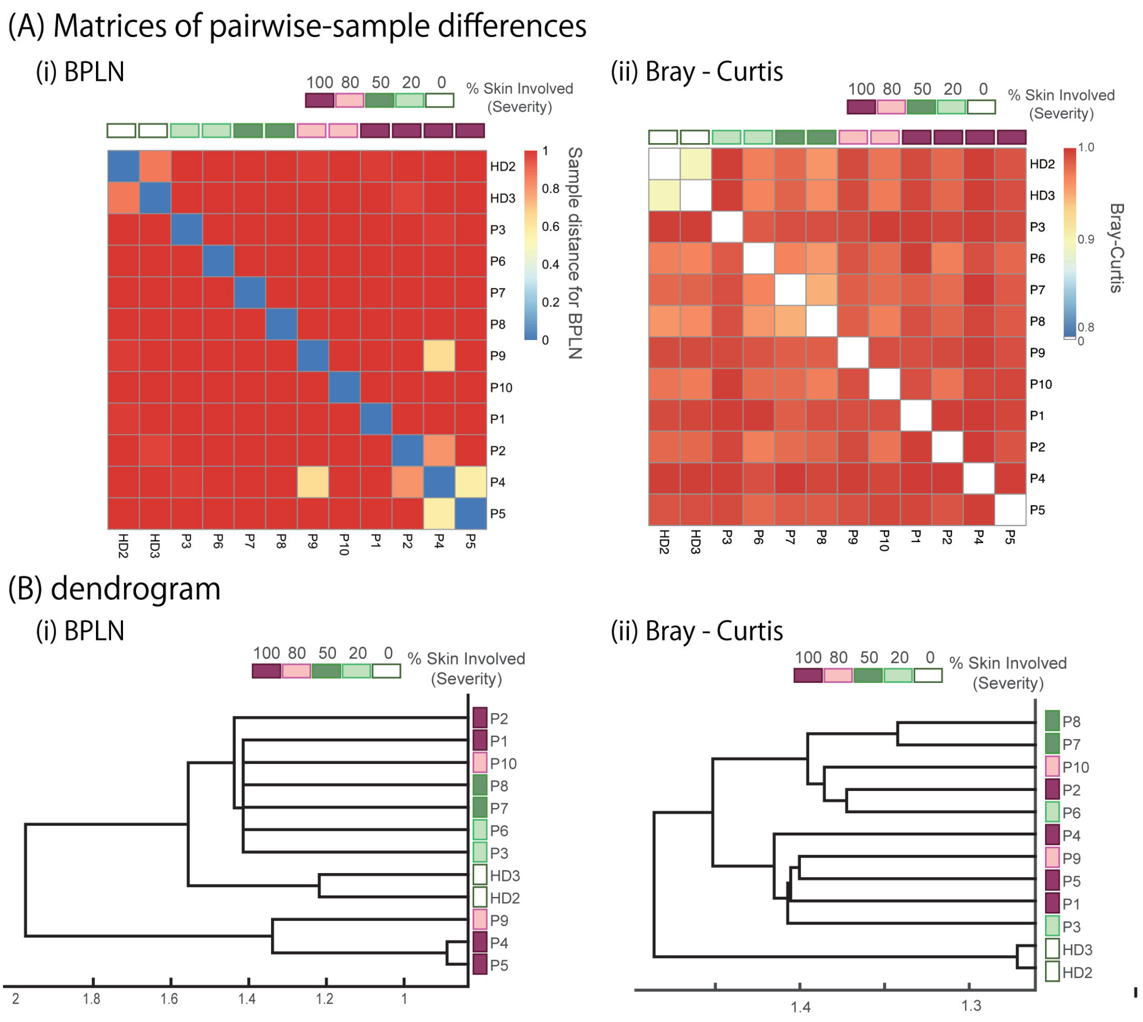

Supplementary Figure 2. Results of applying our methods to the dataset of the human TCR α-chain CDR3 sequences derived from the peripheral blood T cells adopted from the two healthy donors (HD2,HD3) and the ten Sézary syndrome patients (P1-10).
(A) Matrices of pairwise-sample distances and (B) the dendrogram constructed from the matrices.

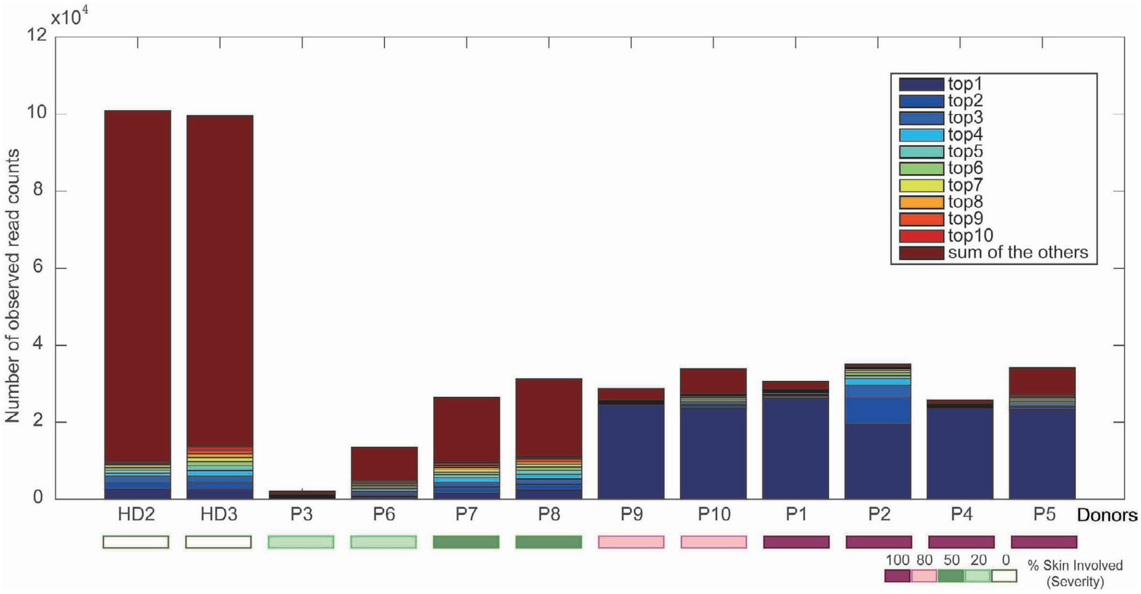

Supplementary Figure 3. Number and relative frequencies of the observed CDR3 sequences. The colors in each barplot indicate the relative frequencies of top 10 observed sequences and the others.

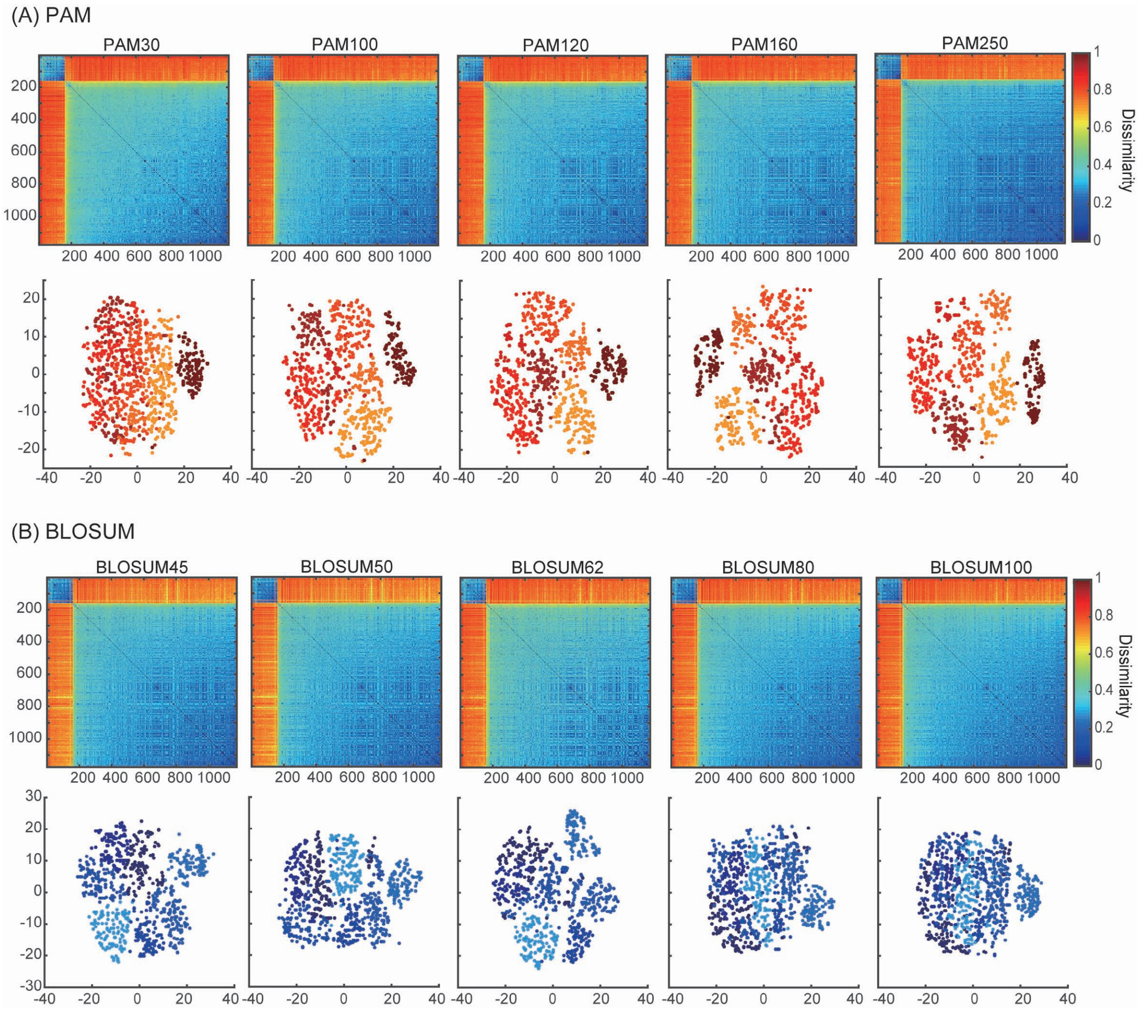

Supplementary Figure 4. Dissimilarity matrices and their embedded distributions calculated by the version 0.16.1 of Scikit-learn, which correspond to Fig. 1 in the main text.

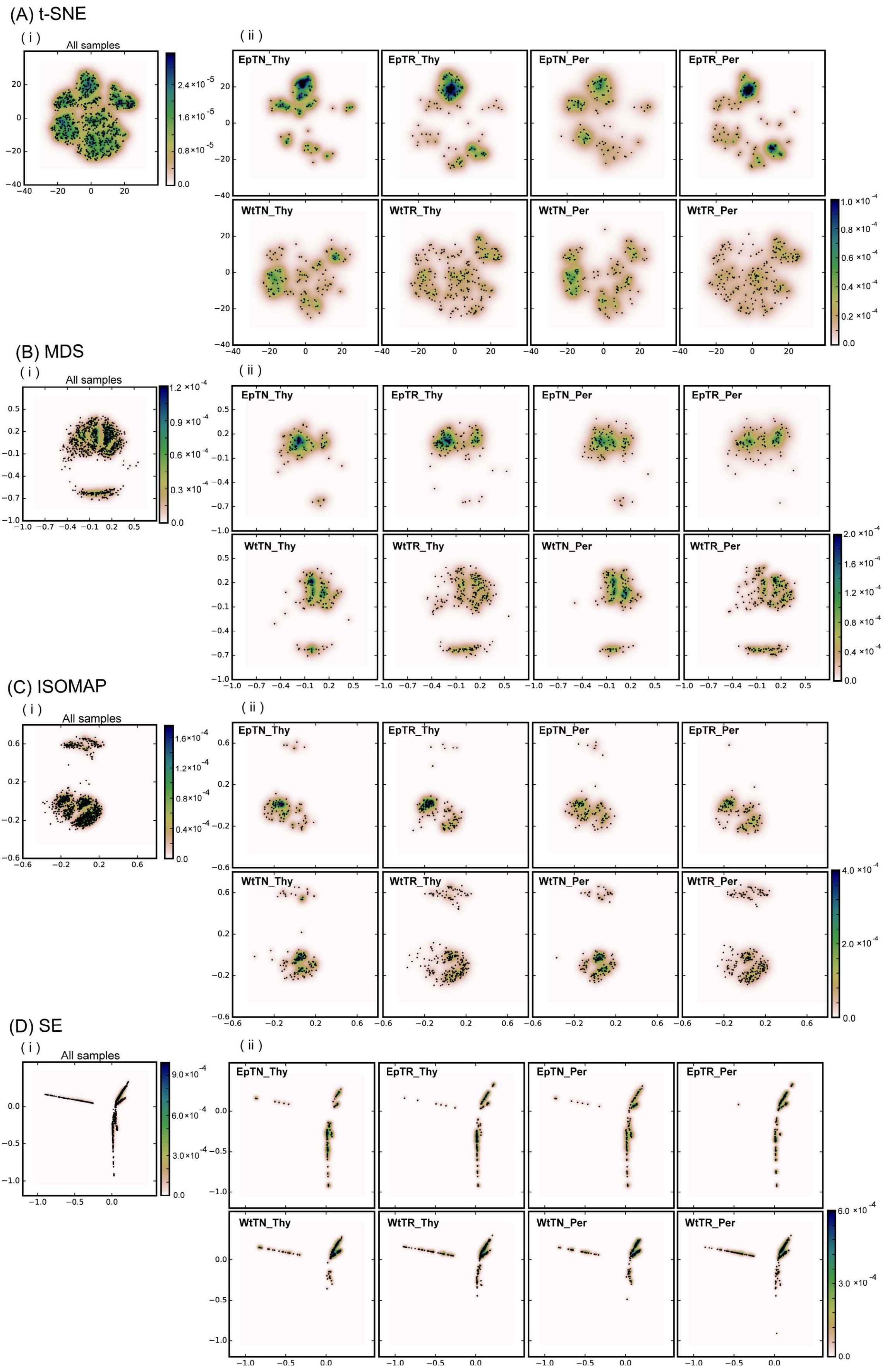

Supplementary Figure 5. Dimensional reduction with the version 0.16.1 of Scikit-learn, which corresponds to Fig. 2 in the main text.

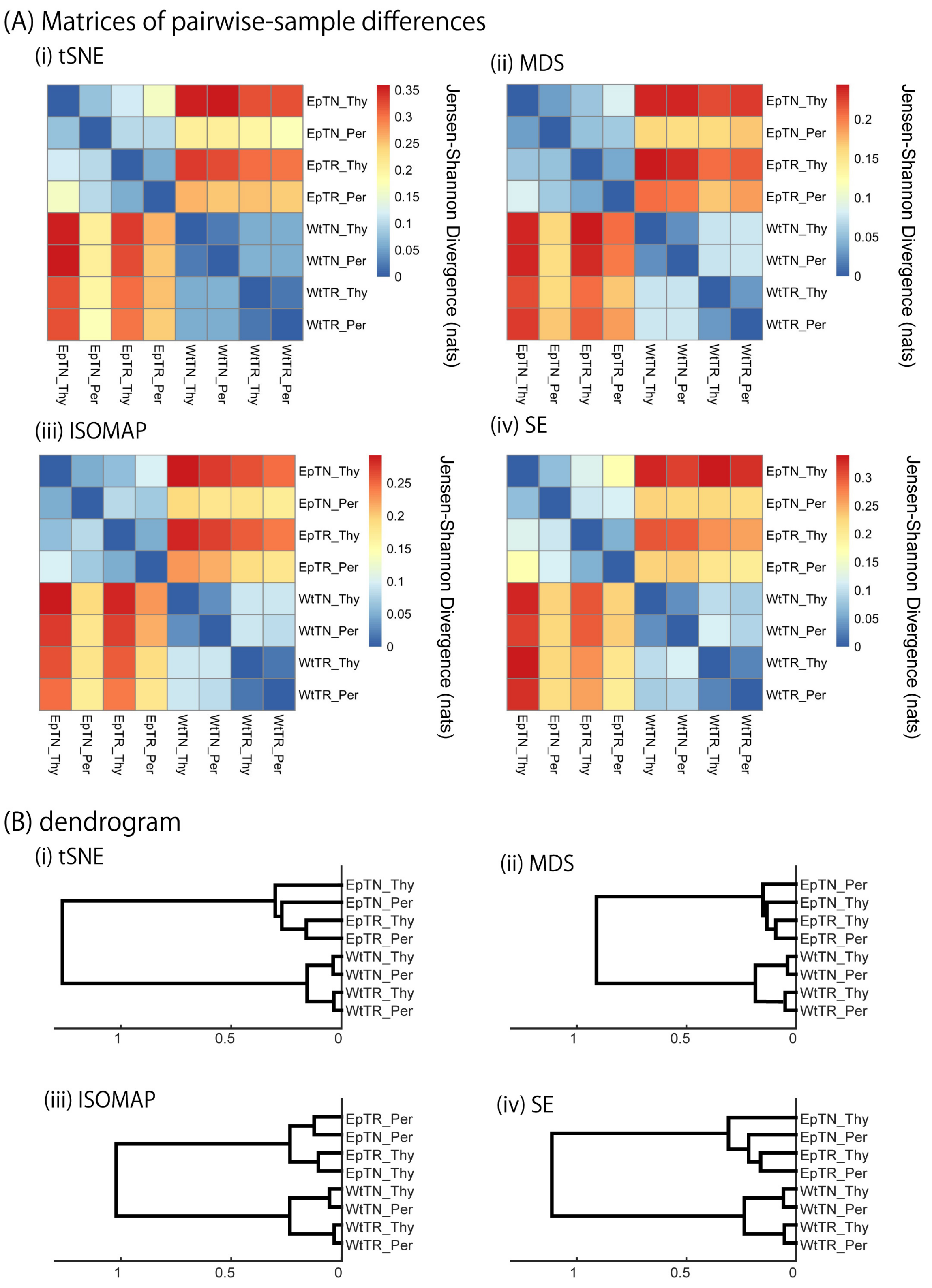

Supplementary Figure 6. JSD matrices and their clustering results with the version 0.16.1 of Scikit-learn, which correspond to Fig. 3 in the main text.

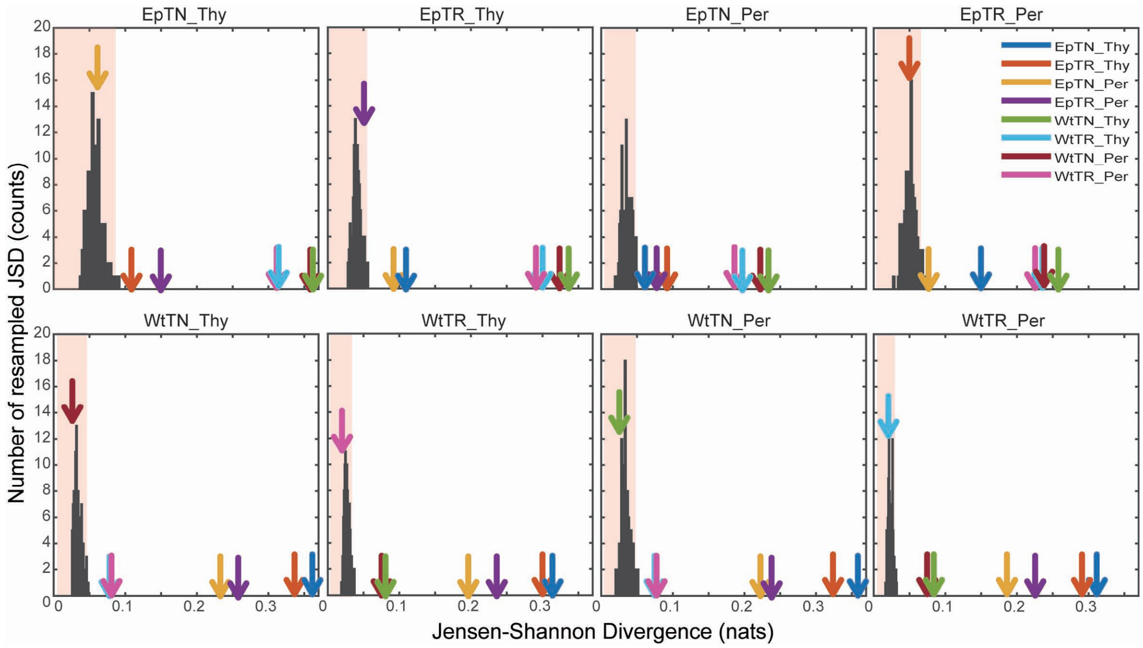

Supplementary Figure 7. Significance tests of JSD values using bootstraps with the result of Fig. S5(A), which correspond to Fig. 5 in the main text.

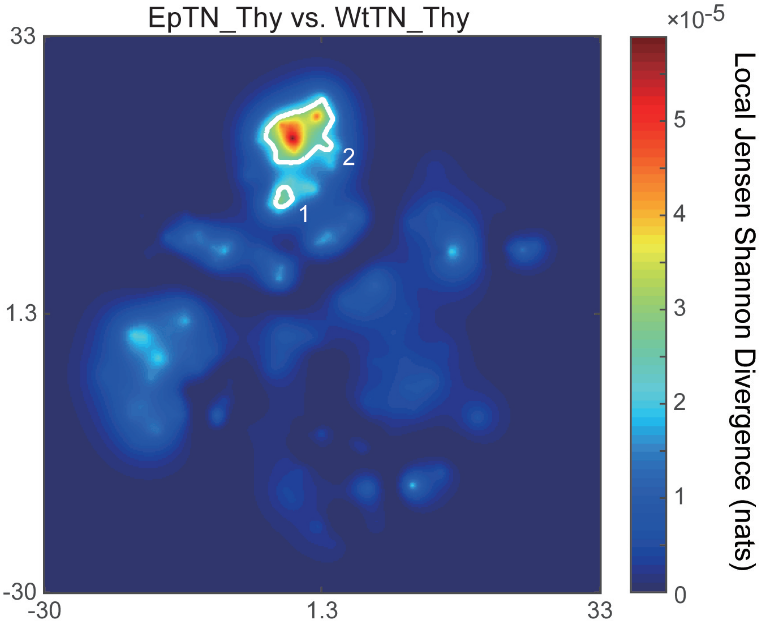

Supplementary Figure 8. Spatial distribution of the local JSD values between EpTN-Thy and WtTN-Thy, which used the result of Fig. S5(A) and corresponds to Fig. 6 in the main text.

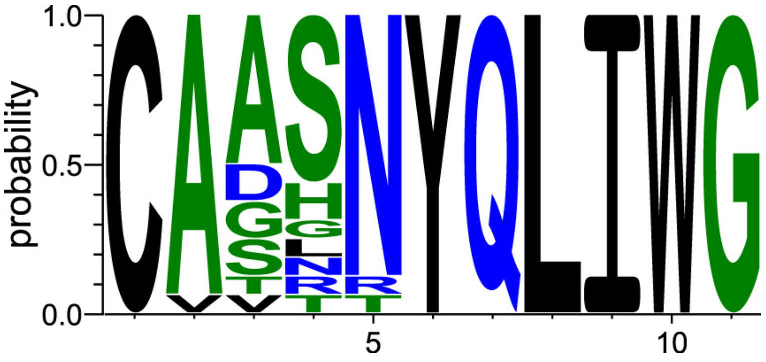

Supplementary Figure 9. Relative frequencies of observed amino acids at each position in the contributing sequences, which used the result of Fig. S8 and correspond to Fig. 7 in the main text.
